# A Review: Adsorption and Removal of Heavy Metals Based on Polyamide-amines Composites

**DOI:** 10.3389/fchem.2022.814643

**Published:** 2022-03-04

**Authors:** Qian Wang, Sining Zhu, Chen Xi, Fan Zhang

**Affiliations:** Key Laboratory of Mineral Cleaner Production and Exploit of Green Functional Materials in Hunan Province, College of Chemistry and Chemical Engineering, Jishou University, Jishou, China

**Keywords:** polyamide-amines, composites, adsorption, heavy metals, waste water

## Abstract

In recent years, the problem of heavy metal pollution has become increasingly prominent, so it is urgent to develop new heavy metal adsorption materials. Compared with many adsorbents, the polyamide-amine dendrimers (PAMAMs) have attracted extensive attention of researchers due to its advantages of macro-molecular cavity, abundant surface functional groups, non-toxicity, high efficiency and easy modification. But in fact, it is not very suitable as an adsorbent because of its solubility and difficulty in separation, which also limits its application in environmental remediation. Therefore, in order to make up for the shortcomings of this material to a certain extent, the synthesis and development of polymer composite materials based on PAMAMs are increasingly prominent in the direction of solving heavy metal pollution. In this paper, the application of composites based on PAMAMs and inorganic or organic components in the adsorption of heavy metal ions is reviewed. Finally, the prospects and challenges of PAMAMs composites for removal of heavy metal ions in water environment are discussed.

**GRAPHICAL ABSTRACT:**
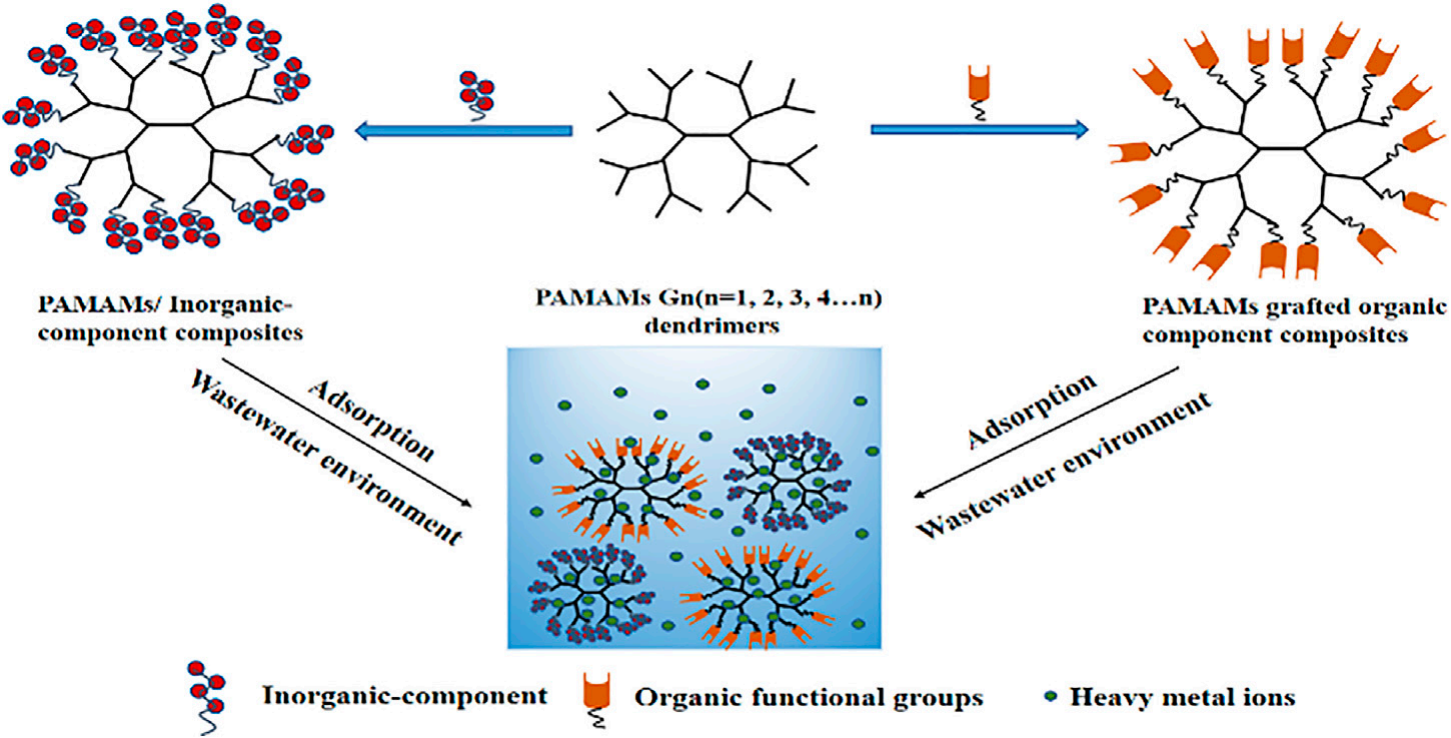


## Introduction

With the rapid development of electroplating, metallurgy, mining, textile leather industry and urbanization, a large number of heavy metal ions such as Pb^2+^ As^2+^, Cd^2+^, Cr^2+^, and Hg^2+^ are being discharged into the aquatic environment via natural environment and man-made means, which contain not only toxic heavy metal ions, but also radioactive elements (Ma et al., 2017a; Luo et al., 2017; Ling et al., 2020; Surgutskaia et al., 2020). The heavy metal pollutants are different from organic pollutants and can’t be biodegraded, the heavy metal ions present in wastewater pose a threat to the environment and human health if not properly being treated. Therefore, the management of heavy metal ions in the environment and the reduction of their content to the allowable concentration are very noteworthy. At present, the main treatment methods for wastewater containing heavy metal ions include the ferrite method, chemical precipitation, the electrochemical method, the reverse osmosis method, the ion exchange method, the adsorption method, and so on (Zhang et al., 2016a; Chen et al., 2021; Mariana et al., 2021). Among them, both ion-exchange and electrochemical methods have high sensitivity and selectivity, but there are shortcomings such as time-consuming, expensive, limited equipment and so on. The adsorption method (Shih et al., 2019; Yuan et al., 2021) has the advantages of large adsorption capacity, fast adsorption speed, recycleability, simple operation, but the preparation process of some heavy metal adsorption materials is complex and the cost is expensive. It can be seen that the existing heavy metal treatment technologies have some deficiencies in one or more aspects (Li et al., 2021). Therefore, it is desirable to consider a method with maximum removal efficiency for heavy metal ions. In previous studies, different kinds of functional groups have been used to construct novel adsorbents (Weidman et al., 2017). Dendritic polymers have attracted great interest in environmental remediation, nanoparticle synthesis, and nano-medicine applications due to their excellent chelating properties (Astruc and Chardac, 2001; Myers et al., 2011). Among them, the most widely studied is the low-toxic polyamide-amine dendrimers (PAMAMs) (Lard et al., 2010). It was first reported in the 1980s and was also the first type of commercial dendritic polymer. Applications of PAMAMs were initially focused on generations with flat ellipsoid shapes (Liu et al., 2005). However, the higher generation of dendritic macromolecules (4 and up) are starburst shaped and logically extended, making them particularly attractive for modern applications (Valdés et al., 2016). At present, there are two main diverse synthesis strategies to synthesize dendritic polyamide-amine with clear structure, namely the convergent growth and divergent growth methods (Hawker and Frechet, 1990; Tomalia, 2005; Tomalia et al., 2010), both of which need to accurately control the growth of its spatial molecular weight. Meanwhile, a divergent convergence method and solid-phase synthesis technologies are both in continuous development (Swali et al., 1997). Some studies have shown that heavy metal ions have a strong affinity with compounds containing amino groups (Aguado et al., 2009; Vasiliev et al., 2009). Since PAMAMs have a large number of terminal amino groups and high specific surface areas, they are considered to be the best choice for forming complexes with metal ions, which makes them be more and more used in the environment. However, the chelate formed by PAMAMs and metal ions has good solubility, which limits its application for the treatment of heavy metal ions (Song et al., 2017). PAMAMs and their modified composites have shown excellent performance in adsorbing environmental pollutants such as heavy metal ions, along with organic and composite pollutions adsorption. Therefore, PAMAMs are used as the main functional groups to prepare the composites with both inorganic and organic components. On the basis of ensuring the structure of the original adsorbent, improving the chelating capacity of heavy metal ions, and achieving the easy separation and recovery after adsorption, which has become an important technical direction in the design of the material for the removal of heavy metal ions.

In this paper, the preparation of the composites based on PAMAMs and inorganic functional components or organic functional groups and their application to the adsorption of heavy metals are reviewed in detail. Finally, future perspectives and challenges encountered with polyamide amines are stated and elaborated, and recommendations are provided for further study.

## The PAMAMs/Inorganic-Component Composites and Their Adsorption for Heavy Metals

### PAMAMs/Carbon-Based Composites

#### PAMAMs/Graphene Oxide Composites

**FIGURE 1:**
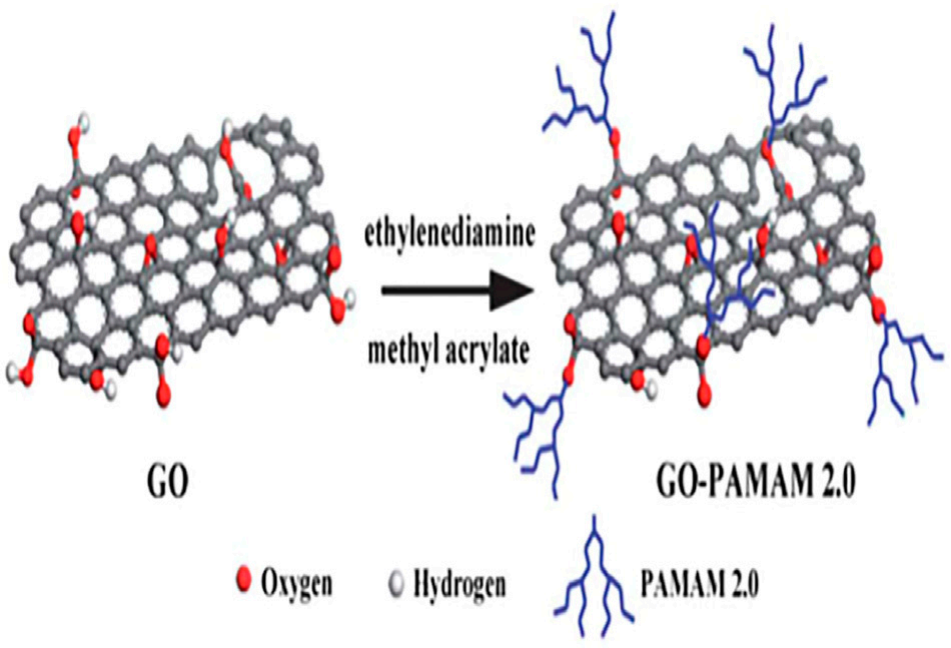
Illustration of the preparation of GO-PAMAM 2.0.

Graphene oxide (GO) is a kind of graphene-based material. Recently, GO has shown strong adsorption capacity due to its large specific surface area and rich oxygen-containing functional groups, including carboxyl, ketone, epoxy, and hydroxyl, which provides various active sites for the modification and adsorption of target pollutants (Zhang et al., 2011; Zhao et al., 2011). However, GO is difficult to separate from aqueous solutions because of its easy dispersion in water, which is likely to cause secondary pollution. Therefore, GO is further functionalized for the purpose of improving its surface properties, so as to facilitate separation and improve adsorption efficiency. Several strategies have been applied to address these extant problems, such as the modification of GO with polymers containing N, S and O functional groups to improve the type and number of functional groups on the GO surface, thereby increasing the ability of GO for removing heavy metal ions (Kong et al., 2021). Table 1 presented some research cases about the PAMAMs/GO composites and their adsorbtion of heavy metals (Pb^2+^, Cu^2+^, Hg^2+^, Mn^2+^, Cd^2+^, Cr(VI), etc.). Obviously, compounding with PAMAMs is a good strategy. For example, Xiao et al. (2016) prepared different generations of PAMAMs functionalized GO (GO-PAMAM) to adsorb Se (IV) and Se (VI) from aqueous solution, Lotfi et al. (2019) designed a new synthesis strategy to investigate dithiocarbamate (DTC) grafted magnetic GO (DTC-D-MGO), the DTC-D-MGO was used as adsorbent for extraction of heavy metals in food and water samples. The GO was modified with PAMAMs *via* a grafting-from method (as shown in Figure 1.) (Yuan et al., 2013). The magnetic GO composites (mGO) were grafted with the first and second generation of polyamidoamine dendrimers and showed their superior adsorption performance for heavy metal ions (Einollahi Peer et al., 2018). Ma (Ma et al., 2017b; Ma et al., 2018) prepared a low generation hexamethylenediamine type polyamide-amine dendritic macromolecule (HMGO-PAMAM-G1.0) with longer hydrophobic chain length by Michael addition and amidation condensation reaction, which exhibited a higher adsorption capacity for Pb(II) and Hg(II) heavy metal ions at higher concentrations. Kommu et al. (2017) applied molecular dynamics simulation to explore the effect of PAMAMs grafted GO on the dynamical and structural properties of Pb(II) ions.

**TABLE 1:** Heavy metal ions adsorbed by polyamide amine/graphene oxide composites.

Adsorbents	Metal ions	*Q*_e_ (mg/g)	Conditions	Model (adsorption isotherm; kinetics)	References
GO-PAMAM-COO^−^	Pb^2+^	1523.1	298K; pH 7.0; C_0_ = 0.5 mol/L	Langmuir isotherm model	Kommu et al. (2017)
HMGO-PAMAM-G1.0	Pb^2+^	108.06	298K; pH 6.2; C_0_ = 300 mg/L	Langmuir isotherm model	Ma et al. (2018)
	Hg^2+^	288.68	298K; pH 6.2; C_0_ = 300 mg/L	Pseudo-second-order	
	Pb^2+^	568.18	298K; pH 4.5; C_0_ = 6 mmol/L		
GO/PAMAMs	Cd^2+^	253.81	298K; pH 5.0; C_0_ = 6 mmol/L	Langmuir isotherm model	Lotfi et al. (2019)
				Pseudo-second-order	
	Cu^2+^	68.68	298K; pH 4.5; C_0_ = 6 mmol/L		
	Mn^2+^	18.29	298K; pH 4.0; C_0_ = 6 mmol/L		
MGO-PAMAM	Hg^2+^	113.71	298K; pH 6.0; C_0_ = 100 mg/L	Langmuir isotherm model	Ma et al. (2017b)
				Pseudo-second-order	
GO-PAMAMs	Cr^6+^	211.42	313K; pH2.5; C_0_ = 240 mg/L	Langmuir isotherm model	Liu et al. (2019)
				Pseudo-second-order	
mGO2nd-PAMAM nanosheets	Cd^2+^	435.85	298K; pH 7.0; C_0_ = 30 mg/L	Freundlich isotherm model	Einollahi Peer et al. (2018)
	Pb^2+^	326.729	298K; pH 6.0; C_0_ = 20 mg/L	R-P isotherm model	
	Cu^2+^	353.59	298K; pH 7.0; C_0_ = 20 mg/L	Langmuir isotherm model	

**FIGURE 2:**
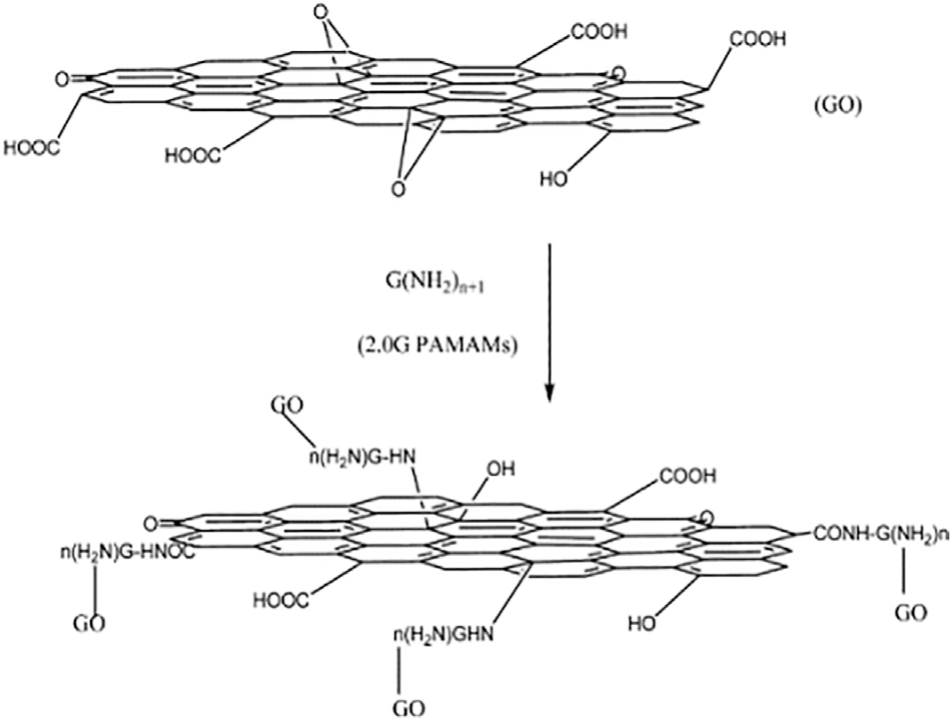
Preparation of GO/PAMAMs composites.

Zhang et al. (2014) prepared graphene oxide/polyamide-amine dendrimers (GO-PAMAMs) by “grafting method” (as shown in Figure 2), the research showed that the adsorption capacity of GO-PAMAMs for heavy metal ions was highly correlated with pH and its maximum adsorption capacity for Pb (II) was 568.18 mg/g (Liu et al., 2019).

#### PAMAMs/Carbon nanotubes(CNTs) Composites

**FIGURE 3:**
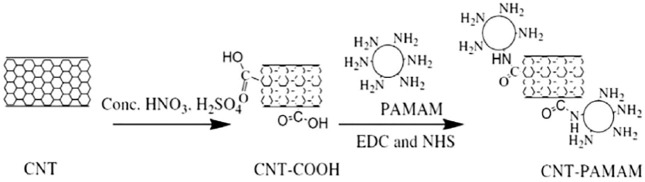
Synthesis process of PAMAM/CNT nanocomposites.

In 1991, IIijima (1991) discovered carbon nanotubes (CNTs) in the cathode deposits of fullerene production by high-resolution microscopy (HRTEM). CNTs are nano-scale hollow tubes made of graphite sheets coiled according to a certain helicity, with a high specific surface area. However, the coagulated suspension of carbon nanotubes in an aqueous solution is caused by the strong intermolecular force between carbon nanotubes, which has a considerable impact on the adsorption performance (Tofighy and Mohammadi, 2011; Ihsanullah et al., 2015; Xie et al., 2015). To improve the suspension concentration of free nanotubes and the adsorption performance of the CNTs, the modification of their surface is the most useful method, which not only enhances the dispersion behavior but also provides more binding sites for the adsorption of metal ions and organic pollutants (Gupta et al., 2016; Hadavifar et al., 2014). For example,Baghayeri et al. (2021) has synthesized PAMAMs covalently functionalized magnetic multi-walled carbon nanotubes for the determination of trace As(III) in an aqueous matrix. The CNTs-coated polyamine dendrimers (PAMAMs) were prepared and used as adsorbents (the composite diagram shown from Figure 3) (Hayati et al., 2017; Hayati et al., 2018), and the results exhibited that PAMAMs-CNT could be effectively used as a novel super absorbent for heavy metal adsorption in multicomponent systems.

#### PAMAMs/Biochar Composites

In recent years, biochar has attracted more and more attention for its unique advantages of a simple synthesis process, low cost, high cation exchange ability, and low environmental impact (Zhao et al., 2017). However, due to the low specific surface area and the limited number of surface functional groups of original biochar, its adsorption and removal efficiency are limited. Thus, original biochar is validly modified to improve the removal performance of contaminants in water by using functional groups that can provide affluent active sites and electrostatic action (Tan et al., 2016; Zhang et al., 2019a; Feng et al., 2020). Yin et al. (2021) prepared a novel magnetic biochar modified with PAMAMs for Cu(II) adsorption, the results indicated that it was an efficient Cu(II) adsorbent and its good recycling performance could also provide a renewable method for resource utilization.

### PAMAMs/Magnetic Nanomaterial Composites

**FIGURE 4:**
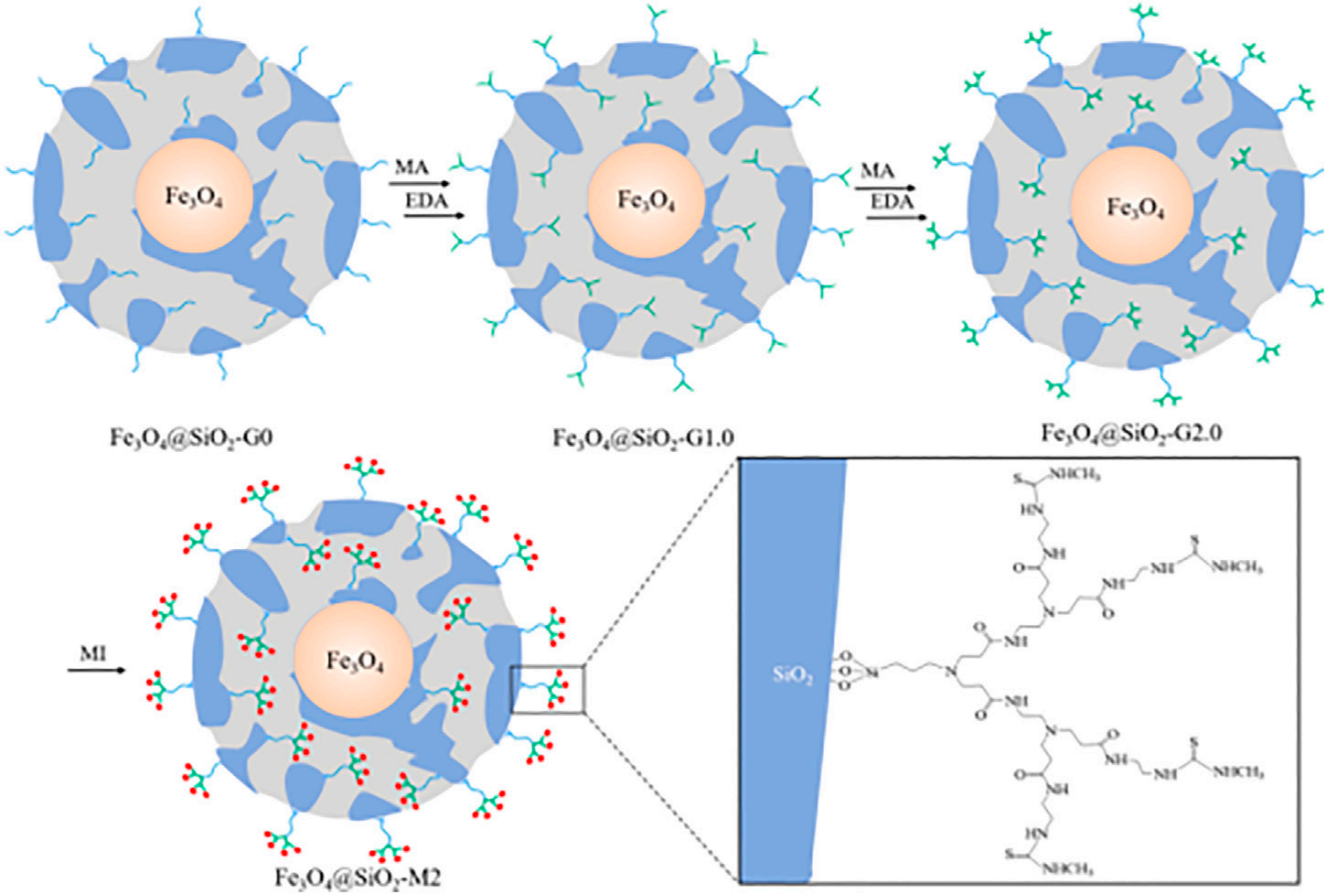
The synthetic procedures for Fe_3_O_4_@SiO_2_-M2.

**FIGURE 5:**
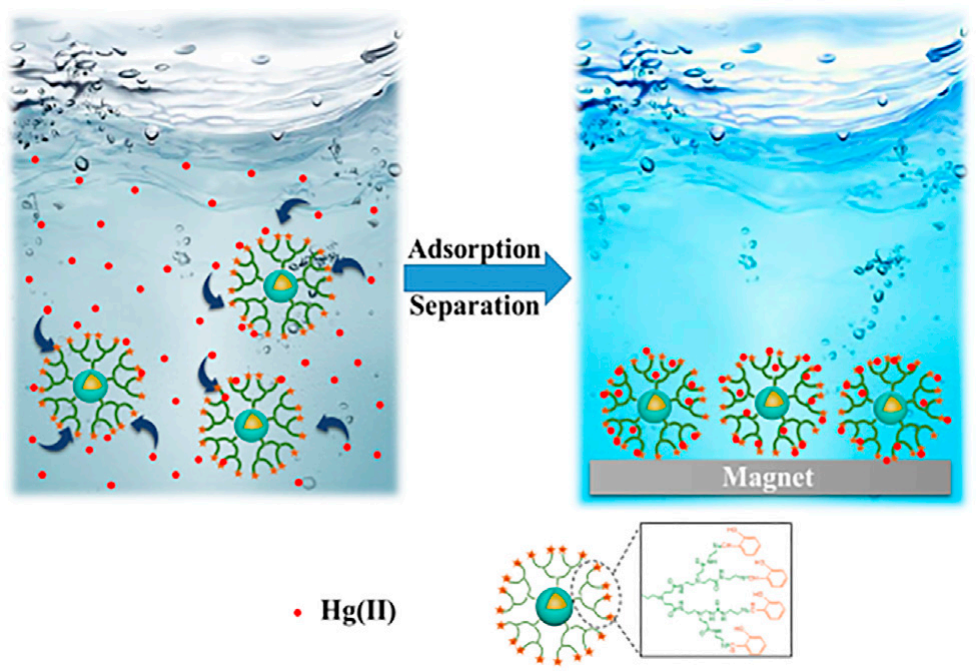
Adsorption mechanism of the Fe_3_O_4_@SiO_2_-G2.0-S.

Great breakthroughs have been made in the development of water treatment technology, and the development and application of adsorption methods are more and more frequent. However, there are deficiencies apparent in the recovery, separation, and reuse of heavy metal adsorbents. The magnetic nanomaterial makes up for these deficiencies to a certain extent owing to the easy phase separation after treatment (Moazzen et al., 2019; Venkateswarlu et al., 2015; Wei et al., 2012). Among many nano-magnetic materials, magnetic Fe_3_O_4_ is the most common and widely used, which can achieve magnetic separation in a short time under the action of an external magnetic field (Zhang et al., 2013). However, bare Fe_3_O_4_ nanoparticles show poor adsorption performance and chemical stability, which limits their application in the remediation of heavy metal ion contamination. Some research published had reported that the modification of the surface of magnetic nanoparticles played a significant role to improve their stability and adsorption properties. Synthesis of organic-inorganic nano-composites is a common method to modify the surface of magnetic nanoparticle (Wang et al., 2010; Liang et al., 2017; Kido et al., 2012). The heavy metals (Pb^2+^, Cu^2+^, Hg^2+^, Ag^+^, Cd^2+^, etc.) adsorbed by PAMAMs/magnetic nanomaterials are summarized in Table 2, the PAMAMs/magnetic composites presented good adsorption properties. For example, Luan et al. (2021a) immobilized sulfur functionalized PAMAMs on magnetic Fe_3_O_4_ composites (Fe_3_O_4_@SiO_2_-M2) (as shown in Figure 4) by combining the advantages of the two components, Ag (I) and Hg (II) can be efficiently and selectively captured and its removal rate of Hg^2+^ was always more than 93.22% after four adsorption-desorption cycles. Sun et al. (2018) successfully prepared core-shell nanocomposites (Fe_3_O_4_@PDA@PAMAM) by synthesis of monodisperse Fe_3_O_4_ nanoflower, which had a high adsorption capacity for Cu (II). Wang et al. (2020) prepared macroporous adsorption resin by sol-gel phase separation and grafted it with dopamine (DA) and polyamide-amines (PAMAMs) for heavy metal ion adsorption. The PAMAMs dendrimer/magnetic Fe_3_O_4_ composites were prepared by Zhou et al. (2020), and the adsorption mechanism was shown in Figure 5. The research work not only explored an improved preparation strategy of a synthetic adsorbent based on functionalized PAMAMs dendritic macromolecules, but also provided an adsorbent with potential application prospects for effective removal of Hg (II) from aqueous solution.

**TABLE 2:** Heavy metal ions adsorbed by polyamide amine/magnetic nanomaterial composites.

Adsorbents	Metal ions	*Q*_e_ (mg/g)	Conditions	Model (adsorption isotherm; kinetics)	References
Fe_3_O_4_@SiO_2_-M2	Hg^2+^	160.47	308.15K; pH 6.0; C_0_ = 0.005 mol/L	Langmuir isotherm model	Luan et al. (2021a)
	Ag^+^	139.15	308.15K; pH 6.0; C_0_ = 0.005 mol/L	Pseudo-second-order	
MNPs-G2-Mu	Pb^2+^	232.56	298K; pH 5.0; C_0_ = 200 mg/L	Langmuir isotherm model	Ekinci et al. (2021)
				Pseudo-second-order	
Fe_3_O_4_@PDA@PAMAM	Cu^2+^	97.18	298K; pH 7.0; C_0_ = 80 mg/L	Langmuir isotherm model	Sun et al. (2018)
				Pseudo-second-order	
Fe_3_O_4_@DA@PAMAM	Cu^2+^	209.7	298K; pH 7.0; C_0_ = 80 mg/L	-	Wang et al. (2020)
	Pb^2+^	262.1			
	Cd^2+^	150.2			
CT-HPMNPs	Hg^2+^	72.3	298K; pH 5.0; C_0_ = 20 mg/L	Freundlich isotherm model	Tabatabaiee Bafrooee et al. (2020)
				Pseudo-second-order	
PAMAMG3-Fe_3_O_4_/P (GMA‑AA‑MMA)	U^6+^	395.2	298K; pH 4.5; C_0_ = 150 mg/L	Langmuir isotherm model	Yuan et al. (2016)
				Pseudo-second-order	
PAMAM-MNC	Pb^2+^	333	298K; pH 5–6; C_0_ = 1,000 mg/L	Langmuir isotherm model	Pourjavadi et al. (2016)
				Pseudo-second-order	
Fe_3_O_4_@SiO_2_-G2.0-S	Hg^2+^	605.78	308K; pH 6.0; C_0_ = 0.01 mol/L	Langmuir isotherm model	Zhou et al. (2020)
				Pseudo-second-order	

Obviously, there are still some deficiencies, such as previous researches were found that Fe_3_O_4_ was easy to be oxidized or corroded, which weakened its magnetism. In addition, iron oxide had poor solubility and tended to form iron hydroxide precipitation at low pH value, and it was not easy to form iron oxide gel.

Pourjavadi et al. (2016) polymerized methyl acrylate on the surface of activated magnetic particles and then grafted PAMAMs with methyl acrylate group to prepare magnetic nanocomposites (PAMAM-MNC). In general, the composite adsorbent obtained by modifying magnetic nano-material with PAMAMs has a certain potential application value for the development of heavy metal adsorption research. Ekinci et al. (2021) successfully prepared magnetically separable nanoadsorbents (MNPs-G2-Mu) by covalently grafting polyamide-amine dendrimers onto magnetite nanoparticles coated with 3-aminopropyl functionalized silica, and applied them to the efficient removal of Pb(II) in aqueous solution. Compared with other traditional methods, the magnetic separation technology of the MNPs-G2-Mu nano-adsorbent is a relatively simple, economical, and efficient method. Bafrooee (Tabatabaiee Bafrooee et al., 2020) synthesized carboxyl terminated hyperbranched polyamide dendrimer grafted with core-shell superparamagnetic nanoparticles (CT-HPMNPs) by chemical coprecipitation method, which was used as a new adsorbent to remove Hg(II) from water. The results show that CT-HPMNPs have significant removal potential for Hg (II) ions in industrial wastewater and labeled water. Even after 10 regeneration cycles, the adsorption capacity would not decrease significantly. Yuan et al. (2016) had grown the adsorbent formed by the third-generation-PAMAM dendritic structure on superparamagnetic epoxy polymer composites by soap-free emulsion polymerization procedure to remove U^6+^, and the results showed that there was no significant loss of adsorption capacity after five adsorption cycles. All these reflect that polyamide-amines composite can achieve effective and regenerative effect in the removal of heavy metal ions.

### PAMAMs/Natural Clay Mineral Composites

Natural clay minerals, such as attapulgite (ATP), palygorskite (PAL), kaolinite and halloysite, etc, that can remove various pollutants from water. And sometimes they will be more feasible and cheaper than synthetic nano-adsorbents (Fan et al., 2009; Gibson et al., 2011).

Among the existing clay adsorbent, palygorskite is an aquifer banded magnesium-aluminum silicate mineral with typical one-dimensional structure (Wu et al., 2017). In addition, PAL has a highly ordered pore structure and a higher specific surface area, which makes PAL show great potential in the adsorption of heavy metal ions. However, the general adsorption and ion exchange are non-specific, and the capacity of natural PAL is greatly reduced due to the strong competition of co-existing metal ions for the natural PAL active center. Furthermore, the PAL is easily reunited in solution, which limits the entry and diffusion of heavy metal ions, resulting in the decrease of adsorption performance (Chen et al., 2010; Deng et al., 2013). The organic functional group is processed to form specific adsorption sites on its surface to enhance the adsorption capacity and selectivity of PAL. Thus, PAMAMs were selected to modify the natural PAL. For example, Zhou et al. (2015) designed a range of novel PAMAMs dendrimer functionalized PAL adsorbents, which were used to adsorb Pb (II) in water solution. It showed a high density of amino-terminal groups in water. Zhang et al. (2019b) prepared a new PVDF/hyperbranched nano palygorskite composite membrane, and the experimental results showed that the prepared composite membrane presented a good binding ability to heavy metal ions. Halloysite is an abundant and novel nano-material with excellent properties of high specific surface area, which can be used in heavy metal wastewater treatment (Zhao et al., 2013; Makaremi et al., 2015). Moreover, after absorption, the halloysite can be regenerated by calcination (Hermawan et al., 2018). Halloysite can be modified with PAMAMs. For example, Cheng et al. (2019) loaded polyamide-amines (PAMAMs) on the surface of magnetic halloysite nanotubes (MHNTs) by one-step thiol-acetylene click chemistry and obtained MHNTs-PAMAMs with high-density terminal amine groups for effective adsorption of Pb (II).

**FIGURE 6:**
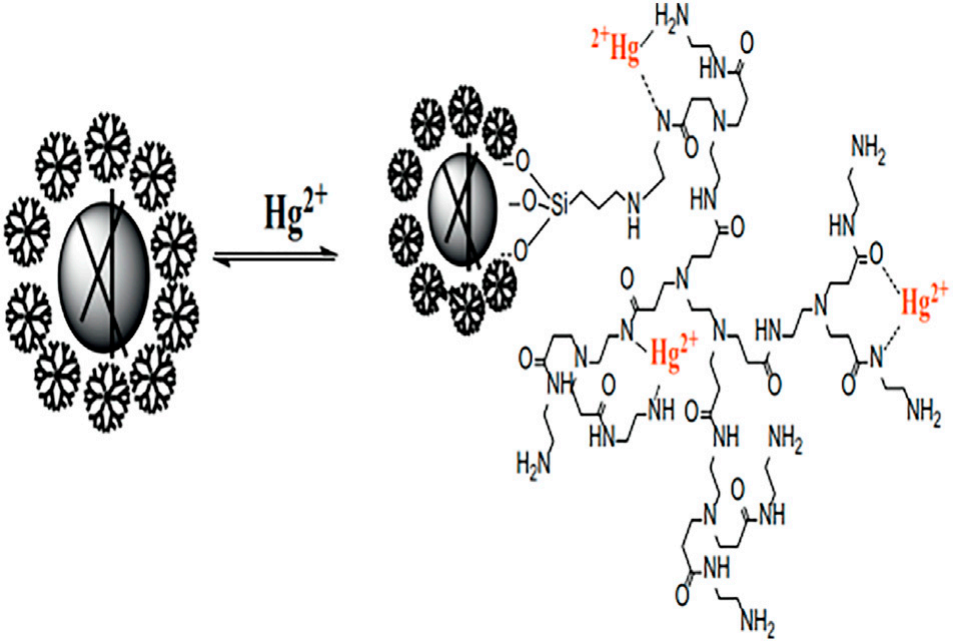
Proposed binding mechanism of PAMAM-ATP with Hg(II).

Attapulgite is 2:1-type layered chain clay minerals with rod-like morphology, large specific surface area, and suitable cation exchange capacity, which can be used in the research field of heavy metal ion adsorption. Due to its excellent stability and good modification properties, it is used as a solid adsorbent and carrier for polymers and organic compounds in many fields (Chen and Wang, 2007; Li et al., 2015). However, ATP crystal bundles are large and difficult to be evenly suspended in a solvent, which greatly limits its application. Therefore, modifying the surface of ATP is a good choice to improve its dispersion performance and adsorption efficiency. PAMAMs have attracted more and more attention because of their terminal amino-functional groups which can enhance the dispersion of ATP and improve the adsorption performance. For example, Qin et al. (2019) prepared a series of PAMAMs modified attapulgite (ATP) adsorbents (G1.0-G4.0 PAMAMs-ATP). The research results showed that the adsorption capacity was still >90% after five cycles, so this material had the potential to be a highly efficient composite adsorbent for Hg^2+^ removal, and the Figure 6 presented the adsorption binding mechanism.

### PAMAMs/Silicon-Based Composites

**FIGURE 7:**
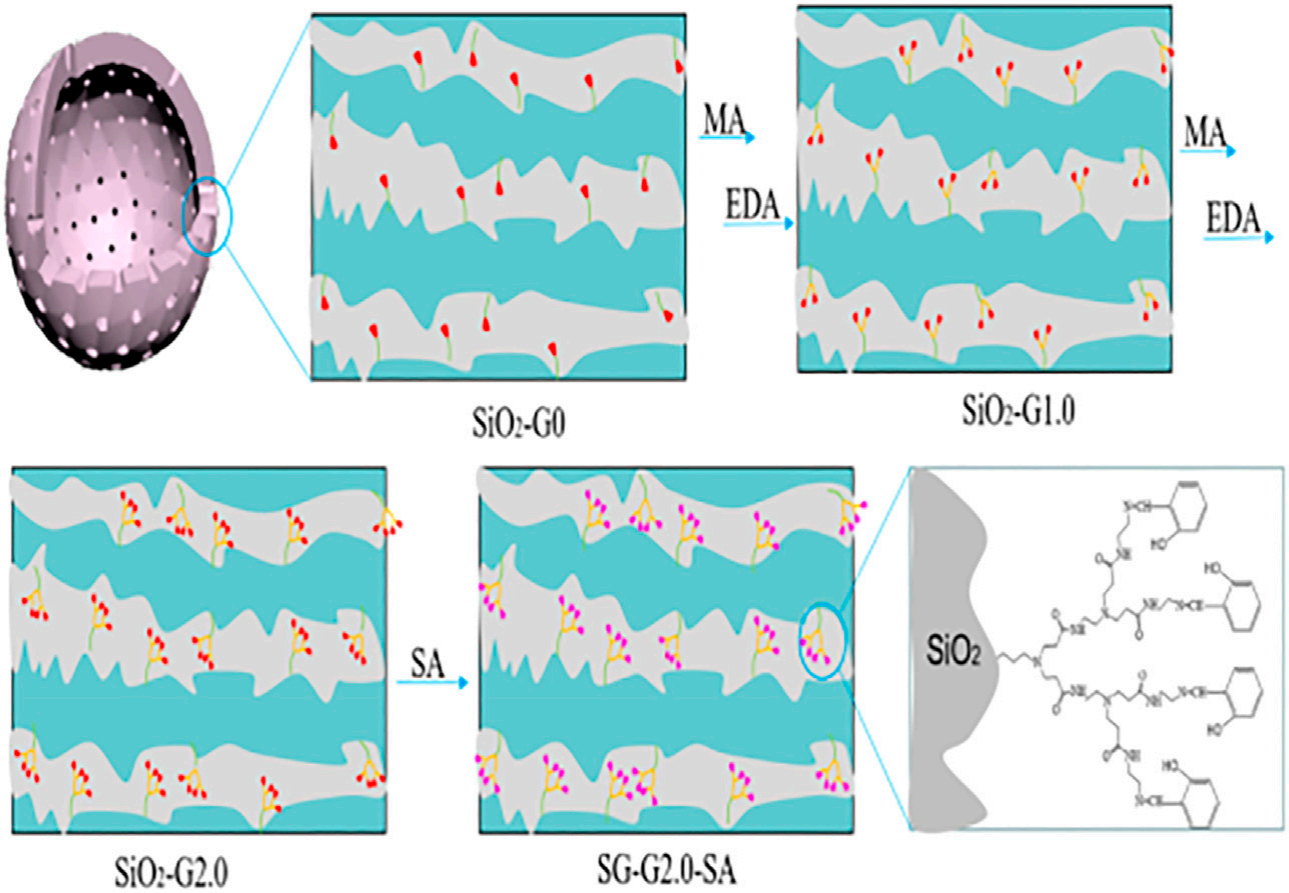
The synthesis route for SG-G2.0-SA.

In recent times, silica has attracted attention in wastewater treatment for its good thermal and mechanical stability, excellent surface functionality, and high specific surface area (Zhao et al., 2018; Lelifajri and Nurfatimah, 2018). A great number of functional groups are used to modify silica to improve its adsorption capacity and selectivity (Kim et al., 2018). These include inorganic-organic hybrid materials, which are inorganic carriers based on the functionalization of organic functional groups, which can also be represented by silica particles grafted by PAMAMs (Diagboya and Dikio, 2018). The functionalized PAMAMs/silica hybrids (as shown in Figure 7) exhibited superior adsorption property than the unmodified PAMAMs (Luan et al., 2021b). At present, polyamide-amine/silicon-based composite materials include the composite with silica gel (Wu et al., 2006; Qu et al., 2008a; Dey and Airoldi, 2008; Kumar and Guliants, 2010; Sun et al., 2011; Radi et al., 2016), mesoporous silica (Sayari et al., 2015), and nano-silica particles (Pawlaczyk and Schroeder, 2019; Qiu et al., 2019; Ren et al., 2019; Ebelegi et al., 2020; Pawlaczyk and Schroeder, 2020; Qiao et al., 2020). The adsorption performance of PAMAMs/silicon-based composites for heavy metal ions (Pb^2+^, Cu^2+^, Ni^2+^, Ag^+^, Fe^3+^, etc.) is summarized in Table 3. These works indicate that the PAMAMs can be used to modify the silicon-based materials to improve their adsorption performance for heavy metals.

**TABLE 3:** Heavy metal ions adsorption by polyamide amine/silicon-based composites.

Adsorbents	Metal ions	*Q*_e_ (mg/g)	Conditions	Model (adsorption isotherm; kinetics)	References
SG-G2.0	Cd^2+^	35.97	313K; pH 5.5; C_0_ = 0.0015 mol/mL	Langmuir isotherm model	Zhu et al. (2018)
	Fe^3+^	13.40	313K; pH 5.5; C_0_ = 0.0015 mol/mL	Pseudo-second-order	
SiO_2_-G2.0-MITC	Hg^2+^	379.12	308K; pH 6.0; C_0_ = 0.004 mmol/L	Langmuir isotherm model	Niu et al. (2016)
				Pseudo-second-order	
SG-MITC-G1.0	Ag^+^	160.71	308K; pH 6.0; -	Langmuir isotherm model	Zhang et al. (2018)
				Pseudo-second-order	
SiO_2_-G2.0	Fe^3+^	37.98	308K; pH 6.0; C_0_ = 0.006 mmol/L	Langmuir isotherm model	Li et al. (2019)
	Ag^+^	70.11	308K; pH 6.0; C_0_ = 0.006 mmol/L	Pseudo-second-order	
PAMAM-n.0SSASG	Pd^2+^	25.05	298K; pH 6.0; C_0_ = 40.0 µg/mL	Langmuir isotherm model	Wu et al. (2016)
				Pseudo-second-order	
SG-G2.0-SA	Zn^2+^	133.43	298K; pH 6.0; C_0_ = 0.0048 mol/L	Langmuir isotherm model	Luan et al. (2021b)
	Ni^2+^	58.69	298K; pH 5.0; C_0_ = 0.0048 mol/L	Pseudo-second-order	
G-3PAMAMSGA	Cd^2+^	28.49	303K; pH 5.0; C_0_ = 175 mg/L	Freundlich isotherm model	Ebelegi et al. (2020)
				Pseudo-second-order	
SiO_2_-dendrimers	Cu^2+^	104.6	301K; pH 5.4; C_0_ = 10 mmol/L	Langmuir isotherm model	Pawlaczyk and Schroeder, (2019)
				Pseudo-first-order	
SiO_2_-PAMAM	Ni^2+^	116.6	298K; pH 5.4; C_0_ = 15 mmol/L	Langmuir isotherm model	Pawlaczyk and Schroeder, (2020)
	Co^2+^	101.1	298K; pH 5.4; C_0_ = 15 mmol/L	Pseudo-second-order	
PAMAM-SBA-15	Cu^2+^	110.58	298K; pH 4.0; C_0_ = 5 mmol/L	Langmuir isotherm model	Shahbazi et al. (2013)
	Pb^2+^	240.35	298K; pH 5.0; C_0_ = 5 mmol/L	Pseudo-second-order	
	Cd^2+^	109.04	298K; pH 5.0; C_0_ = 5 mmol/L		

For example, Zhu et al. (2018) successfully synthesized amino-terminated PAMAMs supported on silica gel and realized the feasibility of removing heavy metal ions in dimethylsulfoxide (DMSO). At the same time, Li et al. (2019) also prepared a series of amine-terminated PAMAMs/silica gel hybrids for the adsorption of Ag(I) and Fe(III) from DMSO. A series of hyperbranched PAMAMs supported by silica was synthesized by Niu et al. (2013), Niu et al. (2014), Niu et al. (2016), Yin et al. (2019), and the adsorption properties for different heavy metals were investigated, such as lead, mercury, chromium, etc., which may be important for the remediation for the heavy metals pollution. Combining the experimental results with DFT calculations, its adsorption mechanism was explored. Zhang et al. (2018) prepared sulfur crown PAMAMs on silica gel, which could be used to adsorb Ag(I) in water. Wu et al. (2016) prepared dendrimer polyamide salicylaldehyde modified silica gel PAMAM-n.0SSASG (*n* = 1, 2, 3, 4), and explored the performance of PAMAM-n.0SSASG microcolumn for separation and enrichment of Cd(II). Jiang et al. (2007) successfully prepared G4-PAMAM-SBA-15 that showed high selectivity for Cr^3+^, Pb^2+,^ and Zn^2+^. Shahbazi et al. (2013) also prepared the functionalized SBA-15 mesoporous silica with polyamidoamine groups, which was used as an adsorbent to remove Cd (II), Pb (II), and Cu (II) ions from aqueous solutions, the research showed that the intermittent and column systems had distinct features, which can be further used to design adsorption systems to purify wastewater contaminated by heavy metal ions.

In general, the prepared PAMAMs/silicon-based composites are good adsorption material, which provides a good application prospect for heavy metal ion adsorption research. However, there are many factors that affect the adsorption materials. In the existed reports, the difference of adsorption of different ions by an adsorbent has not been explained in depth. The influence of the solvent system on the adsorption process still needs further research, and there is no report on the results of combining kinetic and thermodynamic data.

### PAMAMs/Titanium Dioxide Composites

Porous titanium dioxide (TiO_2_) is considered an ideal skeleton material for removing heavy metals because of its excellent chemical stability, low cost, versatility, non-toxicity (Correa et al., 2015; Hallam et al., 2021), and poor hydrolysis stability (Gewert et al., 2015). Functionalization of the surface titania via various anchoring groups for improving adsorption performance of heavy metals, such as silane, carboxylate, amino has been previously demonstrated (Pujari et al., 2014). Barakat et al. (2013a), Barakat et al. (2013b) immobilized G4.0 PAMAMs on titania to obtain novel metal chelation materials for removing heavy metals from aqueous solutions. Maleki et al., 2016) prepared TiO_2_ nanoparticles modified by G4.0 PAMAMs to remove heavy metals in industrial wastewater, and selected metal ions Cu(II), Pb(II), and Cd(II) as model pollutants. The results showed that the hybrid material was a good absorbent with high adsorption performance.

## The PAMAMs Composites Grafted Organic Functional Groups and Their Adsorption for Heavy Metals

### PAMAMs Grafted Organic Small Molecular Materials

The surface modification of PAMAMs by organic small molecules has attracted more and more attention due to the common elements such as alkaline and alkaline earth metals. For example, Zhou et al. (2019) prepared PAMAMs gel through PAMAMs and epichlorohydrin cross-linking reaction, and the maximum adsorption capacity for Cr(VI) got to 267.4 mg/g. Ilaiyaraja et al. (2013a), Ilaiyaraja et al. (2013b), Priyadarshini and Ilaiyaraja (2019) prepared three novel chelating adsorbents (PAMAMG3-SDB, Diglycolamic acid (DGA)-PAMAM-SDB, and PAMAMG3), which were grown on the surface of styrene-diethylbenzene (SDB) by divergent polymerization and used as adsorbents for removing simulated nuclear liquid waste contained Th(IV) and U(VI), and the results showed that these chelating materials could be used as potential adsorbents. In the same year, xanthate functionalized dendrimers were synthesized by Ilaiyaraja et al. (2013c) for removing Co(III) ions from aqueous solution. Hernández Ramirez et al. (2015) prepared a novel metal-chelating porphyrin-PAMAM via the microwave method for removal of hazardous heavy metal ions. The results showed that the removal rate of Pb(II), Cr(II), and Fe(II) by the third-generation porphyrin PAMAMs could reach 99%. In 2003, Rether and Schuster (2003) synthesized novel metal-chelating dendrimers from PAMAMs modified by benzoylthiourea, which can effectively remove heavy metal ions [Co(II), Pb(II), Hg(II), Ni(II), Cu(II), and Zn(II)]. Su et al. (2012) prepared a series of PAMAMs dendrimer hydrophobic ionic liquid N-butyl-N-methylpyrrolidinium bis((trifluoromethyl)sulfonyl)-amide modified with polypyridine-type flexible small molecular ion-carriers or two rigid ionophores to extract Cu (II) ions from aqueous solution. However, the problem of poor selectivity of polypyridine-type modified PAMAMs remains to be solved.

### PAMAMs Grafted Organic Polymer Materials

#### PAMAMs Grafted Synthetic Polymer Materials

**FIGURE 8:**
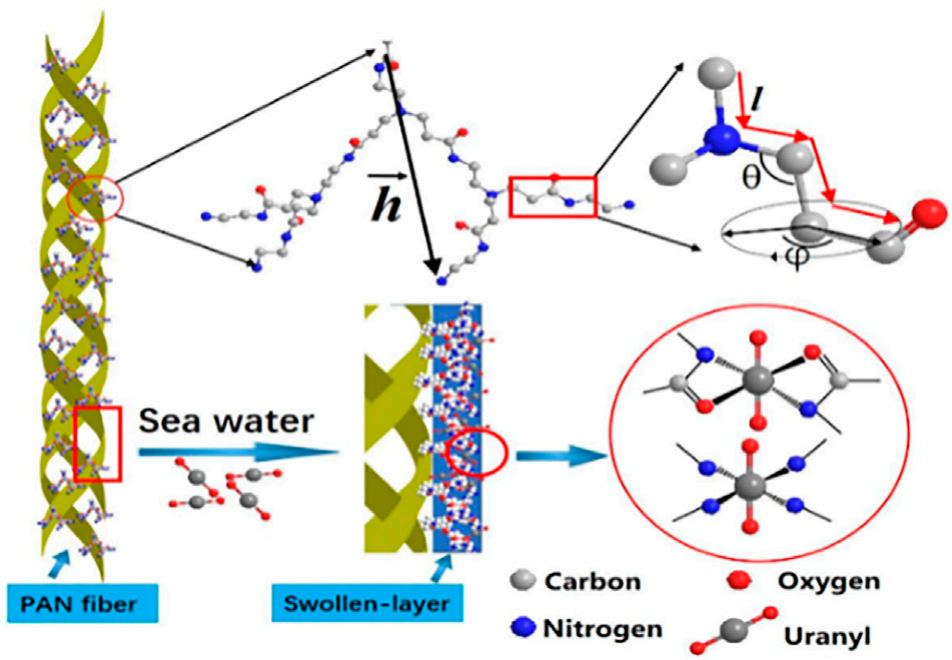
Adsorption mechanism of U(VI) by PAN-nG-PAMAM.

Among all kinds of adsorbents, polymer materials are widely used because of their rich raw materials, changeable product types, high functional group content, easy separation, and easy preservation, and play an important role in the removal of heavy metals. Therefore, there are many studies on their modification, and the heavy metals (Cu^2+^, Hg^2+^, Ni^2+^, etc.) adsorption by polyamide-amines grafted organic polymer materials are summarized in Table 4. For example, Ju et al. (2019) had grown the hyperbranched PAMAMs molecular chain on the surface of polyacrylonitrile (PAN) to obtain the active center adsorbent of PAN-nG-PAMAM (*n* = 1.0, 1.5, 2.0, 2.5, 3.0) polyamine, and extracted U(VI) from seawater. The results illustrated that the maximum adsorption capacity of the adsorbent reached 555.5 mg/g and the adsorption mechanism of U(VI) by PAN-nG-PAMAMs is shown in Figure 8. The PAMAMs modified poly (styrene-co-divinylbenzene) (PS-PAMAM-PPA) was used to adsorb U(VI) from aqueous solution (Cao et al., 2013) and the adsorption and desorption researches indicated that it could be reused 27 cycles. Liu et al., 2014) prepared low-generation polyamide-amines chelating resins (PS-PAMAM-IDA) to preconcentrate Ni^2+^ from synthetic aqueous samples. The study confirmed the feasibility of the chelation reaction between nickel ion and synthetic resin. Diallo et al. (2008) had also done relevant research to recover U(VI) from wastewater.

**TABLE 4:** Heavy metal ions adsorption by PAMAMs grafted organic materials.

Adsorbents	Metal ions	*Q*_e_ (mg/g)	Conditions	Model (adsorption isotherm; kinetics)	References
PVDF-g-PAA-PAMAM	Cu^2+^	100.98	298K; pH 5.5; -	Lagergrensecond-order model	Sun et al. (2019)
PAN-3G-PAMAM	U^6+^	555.5	318K; pH 5.0; C_0_ = 500 mg/L	Langmuir isotherm model	Ju et al. (2019)
				Pseudo-second-order	
PS-PAMAM-PPA	U^6+^	99.89	298K; pH 5.0; C_0_ = 100 mg/L	Langmuir isotherm model	Cao et al. (2013)
				Pseudo-second-order	
PS-PAMAM-IDA	Ni^2+^	24.09 ± 1.79	298K; pH 7.0; C_0_ = 200 mg/L	Langmuir isotherm model	Liu et al. (2014)
				Pseudo-first-order	
CTS-1.0	Hg^2+^	526.32	298K; pH 5.0; C_0_ = 2005.9 mg/L	Langmuir isotherm model	Ma et al. (2009)
				Pseudo-second-order	
HPFC	Cu^2+^	155	318K; pH 8.3; C_0_ = 1,200 mg/L	Langmuir isotherm model	Yu et al. (2019)
				Pseudo-second-order	
G4PSt	Cu^2+^	251.19	303K; pH 5.5; C_0_ = 20 mmol/L	Langmuir isotherm model	Zhang et al. (2021)
				Pseudo-second-order	

**FIGURE 9:**
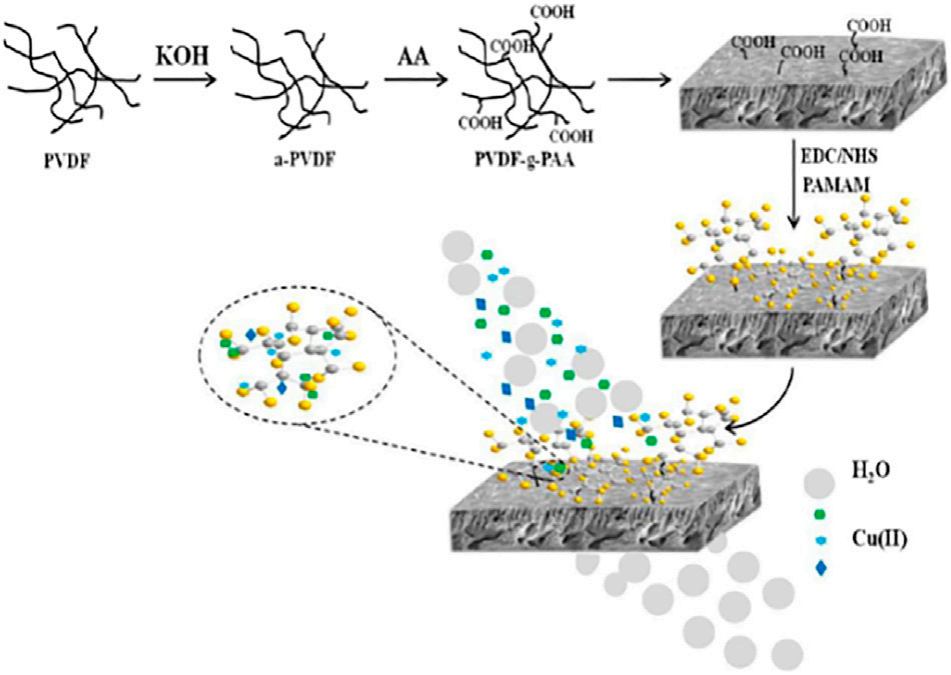
The scheme of preparation of PVDF-g-PAA-PAMAM membrane.

Polyvinylidene fluoride (PVDF) membrane is widely used because of its excellent mechanical strength, acid and alkali resistance, thermal stability, and low cost. However, since the structure of the PVDF membrane does not contain functional groups (-NH_2_, -COOH, -OH, etc.) that can complex with heavy metal ions, the ability to adsorb heavy metal ions is usually improved by introducing some functional groups (Chen et al., 2018). For example, Sun et al. (2019) synthesized PVDF-G-PAA-PAMAM composite membrane by grafting different generations of polyamide-amines onto PVDF-G-PAA membranes (as shown in Figure 9). The composite membrane had good adsorption performance for Cu (II) ions, and the adsorption capacity could reach 100.98 mg/g, which was higher than that of pure PVDF membrane (2.8 mg/g). And after five desorption cycles, the regeneration rate of PVDF-g-PAA-PAMAM membranes was higher than 90%, which had excellent recovery performance. The desorption experiments showed that PVDF-g-PAA-PAMAM membranes had excellent recycling performance. Kotte et al. (2015) prepared a novel mixed matrix polyvinylidene fluoride (PVDF) composites membrane *via* one-pot method, which contained *in situ* synthesized polyamide-amines (PAMAMs) particles, and investigated its adsorption performance for Cu(II). The present experiment shows that the mixed matrix PVDF membranes can act as a novel high-capacity adsorbent to recover Cu(II) from aqueous solution. These studies proved that the modification of PAMAMs was beneficial to the adsorption of Cu(II), laying a certain foundation for the practical application of the PAMAMs modified PVDF membrane in the treatment of wastewater containing Cu(II).

#### PAMAMs Grafted Natural Organic Polymer Material

Chitosan (CTS) is a basic deacetylated product of chitin, which is widely used due to its hydrophilic, non-toxic, easy degradation, and biocompatibility (Chatterjee et al., 2005). A large number of highly active hydroxyl and amino groups exist on the surface of chitosan, which can be used as adsorption sites. However, its low porosity, softness, and tendency to aggregate and form gel has some disadvantages in the treatment process of wastewater (Evans et al., 2002). Physical or chemical modification of chitosan can improve its chemical and mechanical properties. In recent years, the surface chemical modification of chitosan with certain functional groups has attracted extensive attention, because it can significantly improve the stability of chitosan in acidic media and improve its selective adsorption for heavy metal ions (Sun et al., 2006; Donia et al., 2008). Many new functional materials will be obtained by introducing polymer into chitosan. For example, Ma et al. (2009) synthesized the ester and amino-terminated chitosan of dendritic PAMAMs polymer (CTS-1.0–3.0) by divergent method and studied the adsorption behavior of Hg (II) on the adsorbent in their previous work, they also studied the competitive adsorption of different heavy metals (Au^3+^, Pt^4+^, Pd^2+^, Cu^2+^, Ni^2+^, Ag^+^, Zn^2+^, Hg^2+^, and Cd^2+^) on similar compounds, and the results showed that the adsorption capacity of Au^3+^ and Hg^2+^ was stronger than that of other metal ions (Qu et al., 2008b). Zarghami et al. (2016) successfully synthesized different generations of PAMAMs grafted chitosan as a comprehensive biological adsorbent (CS-G0.5∼G5.0) via step by step divergent growth method, and studied the adsorption performance of heavy metals (Pb^2+^) in water. The results showed that the adsorption capacity of CS-G3.0 for Pb(II) was 18 times that of single chitosan.

Cellulose is one of the most abundant natural polymers in the world (Zhou et al., 2013), which has attracted extensive attention because of its richness, sustainability, and easy functionalization. However, the surface area of raw cellulose is not high and the density of binding sites is relatively low, so the adsorption capacity for heavy metal ions is usually not satisfactory (Klemm et al., 2011; Hokkanen et al., 2016; Tao et al., 2017). Therefore, cellulose can be converted into efficient adsorbents by introducing some functional groups with an affinity for metal ions, such as amino, carboxyl, sulfonic and hydroxyl groups (Jin et al., 2017). Existing studies revealed that the composites formed by the combination of cellulose and functional polymers have excellent pollutant adsorption capacity. For example, Yu et al. (2019) synthesized an amino terminated hyperbranched polyamide-amines functionalized cellulose adsorbent for effective removal of dyes and metal ions, and the material shows excellent adsorption for Cu(II). Similarly, Wang and Lu (2019) prepared a series of PAMAMs functionalized nanocrystalline cellulose composites to measure the effect of removing Cu (II) from aqueous solution. Ilaiyaraja (Zhao et al., 2015) synthesized a novel polyamide-amine-grafted cellulose nanofibrils aerogel for the removal of Cr(VI) and the results showed that the maximum removal rate of Cr(VI) reached 377.36 mg/g, which was the highest value of Cr(VI) removal by a biosorbent since it was reported. The modification of corncob and soy hull containing a lot of cellulose in crops was also investigated. Zhang et al. (2016b) prepared PAMAMs functionalized soybean shell (PDFSH) for adsorbing rare earth metal ions (La^3+^, Nd^3+,^ and Sm^3+^) from aqueous solution. Lin et al. (2017), Lin et al. (2018) synthesized a low-cost anionic adsorbent (HPMC) to effectively remove Cr (VI) by modifying corn cobs with hyperbranched PAMAMs, the PAMAMs grafted cellulose had become a very promising adsorbent for the efficient and rapid treatment of some toxic metal pollutants.

Starch is a kind of abundant, renewable, and degradable natural biological material, which can combine with a variety of compounds through physical and chemical actions (Dong et al., 2010). In recent years, modified starch had been used as a heavy metal ion absorbent by introducing substances containing various active groups, such as aminothiazole, acrylonitrile, phosphate, carboxylate, etc (Zhang et al., 2008). For example, Zhang et al. (2021) prepared polyamidomine dendrimer starch with epichlorohydrin as a cross-linking agent, which was used to effectively remove heavy metals and provides a basis for the study of Cu (II) and Zn (II) adsorption.

## Conclusion

PAMAMs can introduce a large number of functional groups, which have achieved good research results in the treatment of wastewater containing heavy metal ions. In the paper, the preparation of the composites based on PAMAMs and inorganic functional component or organic functional groups and its application on the adsorption of heavy metals were reviewed. The adsorption behavior and properties of PAMAMs composites are summarized as follows: firstly, reasonable modification or adjustment of functional groups (including N, O and S functional groups) on the required components is one of the effective strategies to improve the adsorption properties of PAMAMs composites, secondly, the stability of the composite material is improved, and its flocculation effect is improved under acidic conditions. Meanwhile, Langmuir isothermal model and Pseudo-second-order kinetic model can better describe the adsorption data, and most of the adsorption processes are spontaneous and endothermic. In addition, the results of desorption and reuse show that PAMAMs can achieve excellent regeneration effect in removing heavy metal ions.

Although plummy progress has been obtained at this stage, there are still many challenges in the future with the deepening of research. For example, the synthesis process of PAMAMs is complex and the separation process is cumbersome, which limits its further large-scale industrial application in the removal of industrial wastewater. At present, there is still a shortage of numerical models to study the adsorption behavior of heavy metal ions by PAMAMs composites in solution and the molecular interaction mechanism between PAMAMs composites and heavy metal ions are still poorly understood. The effect of solvent system on the adsorption process and its specific adsorption performance during the treatment of heavy metal ions and dye wastewater still needs further study.

The future research direction can be started from the following aspects: the adsorption performance of PAMAMs composites is further improved by optimizing the adsorption reasonably, reducing the cost, simplifying the preparation process and increasing the functionalization pathway. Apart from that, the binding mechanism of PAMAMs composite with metal ions is explored according to the numerical model. All the aspects are the key direction in the future.
